# What naturalistic stimuli tell us about pronoun resolution in real-time processing

**DOI:** 10.3389/frai.2023.1058554

**Published:** 2023-03-16

**Authors:** Magdalena Repp, Petra B. Schumacher

**Affiliations:** Department of German Language and Literature I, Linguistics, University of Cologne, Cologne, Germany

**Keywords:** reference, demonstratives, prominence, perspective, ERPs, comprehension, experimental pragmatics

## Abstract

Studies on pronoun resolution have mostly utilized short texts consisting of a context and a target sentence. In the current study we presented participants with nine chapters of an audio book while recording their EEG to investigate the real-time resolution of personal and demonstrative pronouns in a more naturalistic setting. The annotation of the features of the pronouns and their antecedents registered a surprising pattern: demonstrative pronouns showed an interpretive preference for subject/agent antecedents, although they are described to have an anti-subject or anti-agent preference. Given the presence of perspectival centers in the audio book, this however confirmed proposals that demonstrative pronouns are sensitive to perspectival centers. The ERP results revealed a biphasic N400–Late Positivity pattern at posterior electrodes for the demonstrative pronoun relative to the personal pronoun, thereby confirming previous findings with highly controlled stimuli. We take the observed N400 for the demonstrative pronoun as an indication for more demanding processing costs that occur due to the relative unexpectedness of this referential expression. The Late Positivity is taken to reflect the consequences of attentional reorientation: since the demonstrative pronoun indicates a possible shift in the discourse structure, it induces updating of the discourse structure. In addition to the biphasic pattern, the data showed an enhanced positivity at frontal electrode sites for the demonstrative pronoun relative to the personal pronoun. We suggest that this frontal positivity reflects self-relevant engagement and identification with the perspective holder. Our study suggests that by using naturalistic stimuli, we get one step closer to understanding the implementation of language processing in the brain during real life language processing.

## 1. Introduction

Speakers or writers use different referential forms like definite descriptions or pronouns to indicate the referential status of an entity in the current discourse. Especially the investigation of pronouns has received considerable attention in the literature, including experimental and corpus studies. However, empirical studies have so far mostly focused on examining highly controlled two-sentence items involving two potential antecedents. However, the full complexity of natural language use cannot be captured by highly controlled designs (Alday et al., 2017). Thus, it remains an open question how referential forms are processed in *larger naturalistic discourse contexts*. The present study seeks to investigate the online processing of German personal and demonstrative pronouns using naturalistic stimuli.

Much research on pronoun resolution has been devoted to English third-person personal pronouns (*he/she/they*), but in recent years, research focusing on other languages as well as other types of pronouns has been conducted (e.g., Kaiser and Trueswell, 2008; Ellert, 2010; Hemforth et al., 2010; Kaiser, 2011a; Colonna et al., 2012, 2015; de la Fuente, 2015; Çokal et al., 2018). For example, studies on Finnish have shown that the personal pronoun *hän* and the demonstrative pronoun *tämä* show different sensitivities in terms of syntactic role and word order/information structure (Kaiser, 2003a,b; Kaiser and Trueswell, 2008). Moreover, the contrast between null and overt pronouns e.g., in Spanish, Italian, Greek, and Turkish suggests complementary functions between these two forms (e.g., Dimitriadis, 1995; Turan, 1995; Alonso-Ovalle et al., 2002; Filiaci, 2010). Similarly divided are functions observed in the contrast between personal and demonstrative forms in Germanic languages such as Dutch and German (e.g., Kaiser, 2011b; Schumacher et al., 2015, 2016).

Our study of German personal and demonstrative pronouns further deals with a major limitation of previous research: namely that many or most experimental designs in psycho- and neurolinguistics rely on traditional techniques and conventions that cannot account for the complexity of the input in naturalistic speech (Alday et al., 2017; Hamilton and Huth, 2020). Accordingly, existing studies investigating the neural correlates of demonstrative pronouns have only been conducted in highly controlled laboratory settings that tested two-sentence items with two potential antecedents (and there have been valid methodological concerns for this decision). This limitation has led to the impression that demonstratives preferentially occur in ambiguous contexts and are used as a means of disambiguation. The focus on controlled two-sentence items further means that the naturalistic “real-life” use of demonstrative pronouns has received little attention. Therefore, our study addresses this limitation by examining stimuli in a more ecologically valid situation. Note however that in “real life,” natural language processing is multi-dimensional and includes speaker-hearer interaction, as well as turn taking, back channeling and the processing of multimodal cues such as gestures, facial expressions and eye contact. The approach to naturalistic stimuli taken in the current study is based on the more limited domain of *natural language perception* (following Hamilton and Huth, 2020).

In the next section, we review the existing research on German personal and demonstrative pronouns, including research on real-time correlates of referential expressions and referential processing in naturalistic settings. We then present our hypothesis about the neural correlates of German demonstrative and personal pronouns in naturalistic stimuli. Subsequently, we present a corpus analysis of a narrative text that then serves as input for an event-related potential (ERP) experiment. Finally, we report the ERP experiment and its results, including a *post-hoc* analysis on perspectival centers that appear to have an impact on the processing of demonstrative pronouns.

### 1.1. Demonstrative pronouns in German

German has two types of demonstrative pronouns (*der/die/das* and *dieser/diese/dieses*), which can be naturally used to refer to animate entities (unlike the English demonstrative pronouns *this* and *that*). The present study focuses solely on the online processing of demonstratives from the *der/die/das* paradigm (hereafter: d-pronouns) in comparison to the personal pronouns *er/sie/es* (“he/she/it”).

D-pronouns differ from personal pronouns in terms of distinct interpretative preferences as well as specific discourse functions. Resolution preferences have been generally discussed with regard to the notion of prominence (Grosz et al., 1995; Schumacher et al., 2015; von Heusinger and Schumacher, 2019). The prominence account relies on three basic definitions: (1) prominence is a relational property that makes one element stand out from a set of peer elements (e.g., the discourse referents in the current discourse), (2) it shifts over time, i.e., the prominence status of a referent can change as a discourse unfolds, for instance a referent may have a low prominence status at time point t1 but is promoted to the most prominent entity subsequently, and (3) prominent referents are structural attractors, e.g., they serve as perspectival anchors or allow for more referential variation (Himmelmann and Primus, 2015; von Heusinger and Schumacher, 2019). In relation to this, previous studies indicate that d-pronouns in German tend to avoid reference to the most prominent candidate, where prominence has been associated with subjecthood (Bosch et al., 2003, 2007), proto-agentivity (Schumacher et al., 2016), sentence topicality (Bosch and Umbach, 2007), order of mention (Bosch et al., 2003), and perspectival centers (Hinterwimmer and Bosch, 2016; Hinterwimmer, 2019). For referentially ambiguous context sentences it has thus been argued that personal pronouns, although less constrained in their referential behavior, usually show a preference for the subject/agent/first-mentioned/sentence-topic. D-pronouns, on the other hand, have shown to prefer the object/patient/last-mentioned/non-sentence-topic. It has been further argued that the biases of d-pronouns are more rigid (reflected in high referential choices), while the personal pronoun behaves more flexibly (Kaiser, 2011b; Schumacher et al., 2015, 2016, 2017; Bader and Portele, 2019). For instance, in example (1), the personal pronoun *er* (‘he') tends to refer to the first referent (REF1), but it could also sufficiently refer to the second referent (REF2), whereas the d-pronoun *der* (“he-DEM”) selects the second referent with a high likelihood.

(1) Der Künstler_REF1_ will den Tourmanager _REF2_ treffen. Er / Der …
*The artist*_REF1_ *wants to meet the tour manager*_REF2_. *He / He-DEM …*

In a newspaper-based corpus study, Bosch et al. (2003) evaluated the grammatical function of the antecedent of personal pronouns and d-pronouns and found that personal pronouns preferentially referred to subject antecedents, whereas d-pronouns preferred non-subject antecedents. In a follow-up study, Bosch and Umbach (2007) considered the influence of information-structural properties on pronoun resolution. The authors argue that d-pronouns reject the discourse topic as antecedent, whereas the personal pronoun preferentially refers to a candidate that has already been established as discourse topic. This approach is consistent with previous findings (cf. Bosch et al., 2003, 2007) in as far as the discourse topic in German is often realized as the subject and prefers a personal pronoun as an anaphoric device.

Hinterwimmer and Bosch (2016) expanded the topic avoidance account to a broader view involving the notion of perspective. They argue that d-pronouns cannot refer to a referential candidate which is the *perspectival center* of the utterance. That is, the d-pronoun rejects the candidate from whose perspective the action or state described in the utterance is presented (Hinterwimmer and Bosch, 2016, 2017). Accordingly, d-pronouns are sensitive to the perspective holder and rank the respective referent higher than the subject or topic. Since they avoid the most prominent referential candidate, they do not refer to the perspective holder. Hinterwimmer (2020) refines the account by analyzing several examples from two novels by Wolf Haas, where he shows that d-pronouns are only used to refer to the main protagonist, who is also the discourse topic, when the narrator is clearly identified as the perspectival center in the respective passages.

In addition to interpretive preferences of personal pronouns and d-pronouns, referential expressions not only establish connections with previously mentioned entities, but also indicate to the addressee which referential status an entity may adopt in the upcoming discourse. This function is called the forward-looking behavior of referential forms and is a reflection of the dynamic nature of prominence management in discourse. Studies suggest that demonstratives (both the *der/die/das*- and the *dieser/diese/dieses*-paradigm) have the potential to change the referential structure of the unfolding discourse in such a way that previously less prominent entities are promoted in their prominence status. Thus, demonstratives show a referential shift potential in the upcoming discourse, whereas personal pronouns signal the maintenance of the current referential structure (Abraham, 2002; Fuchs and Schumacher, 2020).

### 1.2. Real-time correlates of referential processing

In terms of neurophysiological processes, studies identified two main language-related ERP components that are relevant for the processing of referential expressions: the N400 and the Late Positivity component (for an overview see Bornkessel-Schlesewsky and Schumacher, 2016). The N400 component describes a negative amplitude peaking around 400 ms after the onset of the critical word and has been associated with the degree of plausibility and expectedness in numerous previous studies (e.g., Kutas and Hillyard, 1983; Delogu et al., 2019, 2021). The Late Positivity describes a positive deflection around 600–800 ms after stimulus onset and has been discussed to indicate the updating of the mental model (Schumacher, 2009; Delogu et al., 2019, 2021).

Investigating the real-time processing of German pronouns, Schumacher et al. (2015) observed a biphasic N400-Late Positivity effect for d-pronouns relative to personal pronouns following contexts with two morpho-syntactically accessible entities, which is taken as evidence for more demanding processing costs for the d-pronoun relative to the personal pronoun. The authors propose that these two effects reflect expectation-based and forward-looking processes, respectively. First, the observed N400 for the d-pronoun is taken as an indication of processing demands that arise due to the relative unexpectedness of the d-pronoun and possibly the exclusion of the most prominent referential candidate. Second, the d-pronoun functions as a trigger of attentional reorienting, and the Late Positivity is taken to reflect the anticipation of changes in the subsequent referential structure and the corresponding discourse updating costs.

Similar effects have been observed in other studies on reference. Negative deflections around 400 ms have been reported for manipulations of distance between anaphor and antecedent, indicating an influence of first mention and recency across multiple sentences (Streb et al., 2004), and for referential ambiguity during pronoun resolution (Nieuwland and Van Berkum, 2006). Definite expressions have been shown to depend on the form of their antecedent (e.g., Swaab et al., 2004; Brilmayer and Schumacher, 2021) as well as on the degree of givenness in the context (Burkhardt, 2006). Overall, these findings can be interpreted with respect to the referential structure in discourse and form-specific expectations.

Positive deflections around 600 ms have been reported for new discourse entities, which introduce new referents into discourse, hence trigger updating of the mental model (e.g., Burkhardt, 2006) as well as for topic-marked entities that cause a shift in the ranking of the discourse referents and thus lead to the updating of the referential structure in the mental representation (Hung and Schumacher, 2012, 2014; Wang and Schumacher, 2013). The Late Positivity has also been associated with processing difficulty, when more inferential effort is required to integrate a definite expression into the current discourse (Schumacher, 2009; Delogu et al., 2019; Aurnhammer et al., 2021). Overall, these Late Positivity effects can be linked to processing demands during the dynamic updating of the discourse representation.

In language processing and hence in the processing of reference, perspective-taking also plays a crucial role, because the perspectival center is not a fixed property, but can shift in the course of an interaction from the perspective of oneself to the perspective of another person and from the narrator/speaker's viewpoint or inner thoughts to a protagonist's viewpoint or inner thoughts. Studies have shown that interlocutors are sensitive to subtle perspectival cues that are not directly encoded in the morphosyntax of languages such as English and German. It has been assumed that in narratives, perspective-taking follows a speaker-default stance, but it can change to a non-speaker perspective when enough contextual cues are made available to promote the non-speaker perspective (Harris, 2021). Numerous studies have shown that shifting the current perspective elicits cognitive costs (Hanna et al., 2003; Harris and Potts, 2009; Hartung et al., 2016; Harris, 2021). In electrophysiology, studies observed positive deflections for shifts of perspective. For instance, Richter et al. (2020) used Keysar et al.'s (2000) well-known referential communication game to test the influence of common ground information and privileged information. The authors observed an enhanced frontal late positivity for the condition, in which the perspectives of speaker and addressee differed; this required the integration of common ground information while suppressing privileged information (Richter et al., 2020). Other studies investigating theory of mind also found positive-going ERP effects at frontal electrodes: Meinhardt et al. (2011) examined false- and true-believe reasoning in experiments based on the Sally-Anne-task (Baron-Cohen et al., 1985) and observed a late positivity for the false belief condition relative to the true belief condition. Sabbagh and Taylor (2000) investigated theory of mind *via* short stories that created either a mental representation based on the belief of a protagonist or a non-mental representation based on a photo that was taken by a protagonist. The experiment revealed an enhanced positivity for the belief condition where participants had to take the perspective of a protagonist in order to answer the control question, instead of recalling the image of a photograph that was described in the text. Furthermore, self-directed perspective-taking evoked an early positivity effect relative to non-self-directed perspective-taking: first-person pronouns show stronger positive (P300) responses compared to third-person pronouns (Zhou et al., 2010; Shi et al., 2011; Brilmayer et al., 2019). Overall, these studies indicate that shifting the perspective (e.g., from the narrator to a character, from self- to other-reference) elicits a positive deflection with a frontal maximum that can occur in different time windows and intensities depending on the kind of perspectival shift and task demands. It remains an open question, whether perspective-based adjustments contribute to the Late Positivity that has been framed in terms of discourse updating above or evoke a perspective-specific ERP signature. Furthermore, in line with the findings from the N400 discussed above, perspectival mismatches also evoke an N400 effect (e.g., García-Marco et al., 2016).

### 1.3. Referential processing in naturalistic settings

It is important to note though, that most of the above-mentioned studies (except Brilmayer et al., 2019; Hinterwimmer, 2020 and Brilmayer and Schumacher, 2021) used highly controlled items with ambiguous context sentences involving two potential antecedents. Many or most experimental designs in neurolinguistics are still based on classic lab experiments, which have several drawbacks. For instance, many studies use construed and isolated items that can hardly be related to real-life speech processing. Even though these stimuli are often drawn from real-world sources, it is very uncommon in real-life to hear a sentence that is not embedded in some context. Uncontextualized sentences might also lead to a decrease of the participant's intrinsic motivation to comprehend or process the sentences (Hamilton and Huth, 2020). However, using naturalistic stimuli, one has to be aware of the fact that ecological validity (“naturalness”) and experimental control are two extremes on a continuum, so a gain of one leads to a loss of the other (Brilmayer and Schumacher, 2021).

Nevertheless, in psycho- and neurolinguistic research, there is a growing interest in speech and language processing under naturalistic circumstances. By performing experiments using naturalistic language stimuli, with connected sentences that approximate or draw directly from language as it is used in everyday life (Hamilton and Huth, 2020), we get one step closer to understanding the implementation of language processing in the brain during real-life language processing. Highly natural approaches have already been widely used in studies that investigate neuronal processes during natural reading (Kliegl et al., 2012). However, for studies that investigate how the brain understands or processes language in real-time, natural stimuli have found only limited use so far but are becoming more and more popular. At this point, studies investigating German pronoun resolution using naturalistic stimuli are still quite rare. To our best knowledge, there are no studies investigating the neural processing of d-pronouns with naturalistic stimuli. Brilmayer et al. (2019) and Brilmayer and Schumacher (2021) investigated referential behavior including German personal pronouns in a series of studies using the audio book “The Little Prince”; looking at German first-person personal pronouns in comparison to second- and third-person personal pronouns, Brilmayer et al. (2019) found a P300 effect for first-person pronouns that is in line with previous research on self-relevance (e.g., Knolle et al., 2013). They conclude that first-person marking is an attentional feature of self-relevance that is at the core of successful narrative comprehension (Brilmayer et al., 2019). Moreover, they found an effect for pronouns referring to the main protagonist in comparison to pronouns referring to other protagonists in form of a frontal positivity emerging between 200 and 500 ms (Brilmayer et al., 2019). Further, the relationship between the form of a referential expression (pronoun vs. noun) and the form of its antecedent (pronoun vs. noun) was investigated by Brilmayer and Schumacher (2021) revealing an influence of the form of the antecedent expression on the N400 amplitude following an anaphor.

Overall, investigating pronoun resolution in more naturally produced texts appears to be a promising approach to investigate referential processing under more realistic constraints and to foster the continuous construction of a mental representation of the unfolding discourse. In the following study, we thus bring together the desire to investigate language processing in a more realistic setting (i.e., listening to an audio book) with insights from pronoun resolution in the lab.

## 2. Current study

The purpose of the present study is to assess how d-pronouns and personal pronouns are processed in larger naturalistic discourse contexts. To this end, we recorded ERPs while participants were listening to an audio book version of the German coming-of-age novel *Tschick* by Herrndorf (2010). The novel is about an unusual friendship between the 14-year-old Maik Klingenberg and the teenage Andrej Tschichatschow, nicknamed Tschick. Together, the two teenagers drive through the East German provinces in a stolen Lada and experience many adventures. For the current linguistic analysis, the novel is of interest due to three main reasons: First, the novel is characterized not only by a naturalistic and conversation-like narration style, but especially by the very authentic use of youth language. In this respect, *Tschick* includes also very explicit swearwords and verbal insults. Second, the novel is written from the first-person narrator Maik's point of view and thus is characterized by a homodiegetic narrator, which is an interesting parameter for later analyses. Third, the novel consists largely of dialogue structure, which is another factor supporting the naturalistic language used in the novel [see (2)].

(2) «Wenn **die uns** nachläuft, ist megakacke», sagte **Tschick**.
«Das mit dem Stinken hättest **du** nicht sagen müssen.»
«Irgendwas musste **ich** ja sagen. Und Alter, hat **die** voll gestunken! **Die** wohnt garantiert auf der Müllkippe da. Asi.»
«Aber schön gesungen hat **sie**», sagte **ich** nach einer Weile. «Und logisch wohnt **die** nicht auf der Müllkippe.» (Herrndorf, 2010, p. 158)
“*If* ***she (D-Pro)*** *follows* ***us**, that‘s supercrap,” said* ***Tschick***.
“***You*** *didn't have to say the part about (her) stinking.”*
“***I*** *had to say something. And dude*, ***she (D-Pro)*** *really stunk!* ***I***'*m sure* ***she*** ***(D-Pro)*** *lives at that garbage dump. Lowlife.”*
“*But* ***she*** *sang beautifully,”* ***I*** *said after a while. “And obviously* ***she (D-Pro)*** *doesn't live at the dump.”*

Prior to EEG recording, the text was annotated for features of the pronouns and their antecedents. In particular, we were interested in properties of the direct referential chain between a pronoun and its previous antecedent. Note that we consider the antecedent the element that immediately precedes a referential expression and refers to the same extra-linguistic referent. Therefore, we use the term *previous antecedent* to differentiate our definition of antecedent from the broader traditional one (Schwarz-Friesel and Consten, 2011). In particular, we annotated (i) the type of referring expressions (such as personal pronoun, d-pronoun, …), (ii) their grammatical function (subject, direct object, indirect object, oblique) and (iii) their thematical role (proto-agent, proto-patient, proto-recipient, following the typology of Dowty, 1991; Primus, 1999) of all animate referential expressions in the corpus [in (2) the annotated referential expressions are marked in bold]. Subsequently, we analyzed the annotated features of both critical pronouns as well as their previous antecedents because earlier work indicates a strong influence of these features on prominence computation. Studies by Schumacher et al. (2015, 2016) showed that the prominence-lending cues of the antecedent have a strong influence on pronoun resolution of personal and d-pronouns in German. They emphasized the importance of thematic role information, finding that proto-agentivity is a higher-ranked constraint on pronoun processing than grammatical function. On the one hand, the corpus study served to examine more closely the properties of referential expressions in naturalistic texts, paying particular interest to personal pronouns and d-pronouns. On the other hand, the corpus investigation served as a basis for the EEG study.

## 3. The *Tschick* corpus

### 3.1. Annotations

To better understand the referential behavior of personal pronouns and d-pronouns in our corpus, we conducted extensive annotations of the referential expressions. Therefore, we created a corpus that was formed from 9 chapters (chapter 28–31, and chapter 42–46) from the novel *Tschick*. We annotated all animate referential expressions of the selected nine chapters using the web-based multilayer annotation software Webanno 3.6.7 (Yimam et al., 2014). The annotations were carried out by three annotators in parallel. The corpus data was automatically segmented into sentences and tokenized; inconsistencies were manually checked and corrected prior to annotations. The annotation process was as follows: The chapters were always annotated chronologically. In a first step, sentence segments were determined, in order to create a comparable sentence equivalent since the length of the sentences often strongly varies. Second, all animate referential expressions were marked and the features (i) types of referential expression (RE) (personal pronouns, d-pronouns, demonstrative pronouns, proper names, definite DPs, indefinite DPs, coordinated DPs, relative pronouns, resumptive d-pronouns, resumptive personal pronouns, indefinite pronouns, possessive pronouns, possessive proper names, quantifiers, reflexives, or zero pronouns), (ii) grammatical role (subject, direct object, indirect object, or oblique) and (iii) thematical role (proto-agent, proto-patient, or recipient) were assigned. In a last step, referential chains were identified.

### 3.2. Corpus-based analysis

**Figure 1:**
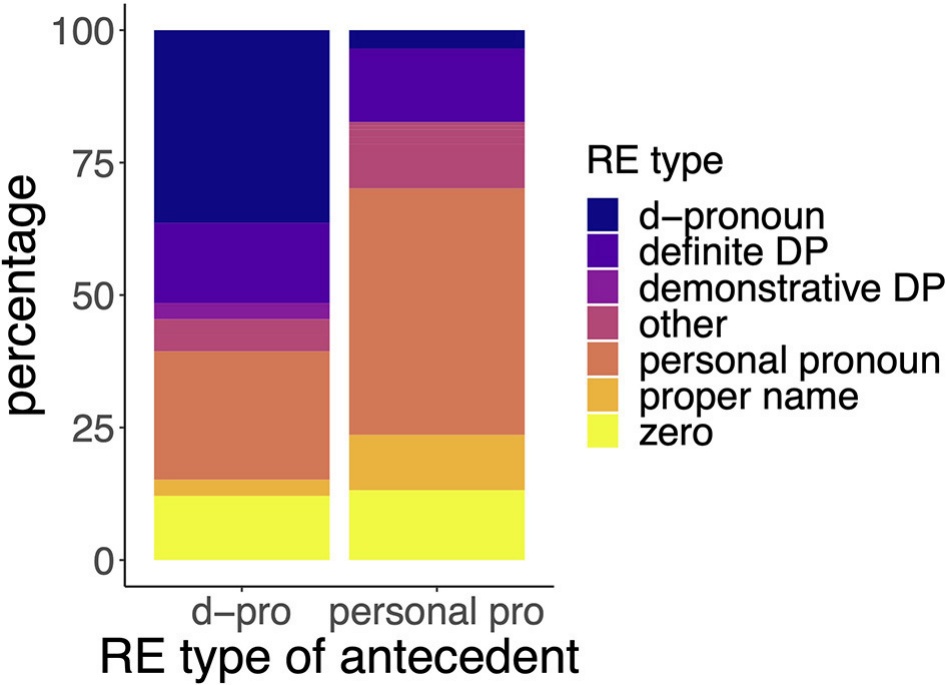
Distribution of type of referring expression of the critical pronoun's antecedent. Distribution of d-pronoun's antecedent on the **(Left)**, distribution of personal pronoun's antecedent on the **(Right)**.

**Figure 2:**
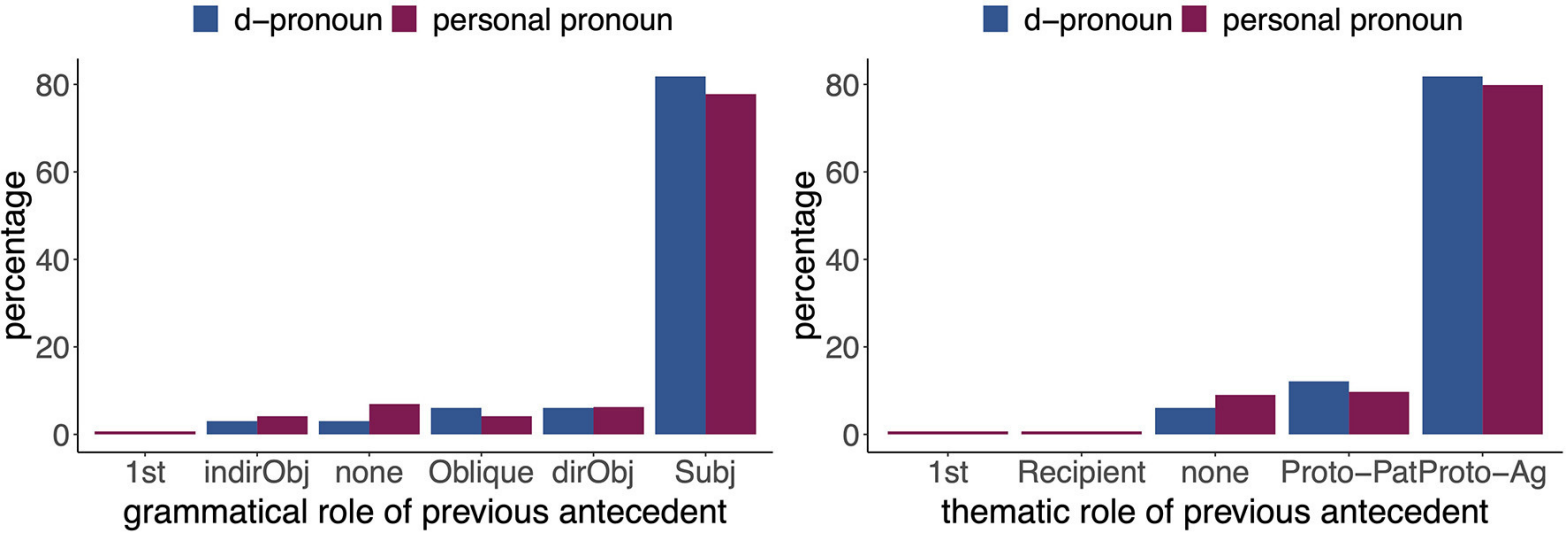
Distribution of grammatical role **(Left)** and thematical role **(Right)** of the antecedents of the personal pronouns (red, *n* = 181) and d-pronouns (blue, *n* = 36).

In total the corpus contains 1,559 animate referential expressions (REs). Out of these, 81.78% are pronominal expressions. These pronominal expressions include among others personal pronouns (53.05% of all REs, *n* = 827), d-pronouns (2.76% of all REs, *n* = 43), possessive pronouns (8.21% of all REs, *n* = 128), and zero pronouns (11.48% of all REs, *n* = 179). Of the 827 personal pronouns, 22.25% (*n* = 184) occurred in third person singular. This rather low count of third person singular personal pronouns is due to the first-person narrator (with 44.86% of pronominal expressions being first person singular pronouns). Of the 43 d-pronouns, 38 occurred in third person singular. Also, we were only interested in feminine and masculine personal and d-pronouns, hence we excluded two neuter d-pronouns and one neuter personal pronoun^1^ as well as three contractions of personal pronouns (*isser* “is-he”). Interestingly, 91.67% of the remaining d-pronouns in third person singular served as the subject and agent of the sentence. Also, 79.56% of the remaining third person singular personal pronouns were the subject and the agent of the respective sentence. Since most personal and d-pronouns occurred in subject and agent position, we subsequently decided to examine only those pronouns in a neurophysiological study, resulting in a total of 33 critical d-pronouns and 144 critical personal pronouns. Looking at the previous antecedents of both the critical personal and d-pronouns, it becomes clear that the two pronoun types behave very similarly in our corpus. Both pronoun types refer to an antecedent that has the same RE type in a considerable number of cases. Personal pronouns refer to an antecedent with the RE type personal pronoun in 46.26% of cases. Similarly, d-pronouns refer to an antecedent that has the RE type d-pronoun in 35.29% of cases. In addition, in 24.24 % of cases d-pronouns refer to an antecedent that has the RE type personal pronoun (see Figure 1). Further, personal pronouns and d-pronouns show similar distributions with regard to the grammatical and thematical role of their antecedents. Both pronoun types mostly refer back to a subject and agent antecedent (see Figure 2). These findings are rather surprising, since many previous studies indicated varying, if not even complementary patterns for d-pronouns relative to personal pronouns with respect to the prominence-lending features of their antecedent (Abraham, 2002; Bosch et al., 2003, 2007; Schumacher et al., 2016). For instance, previous accounts suggested that d-pronouns (as referent shifters) do not refer back to d-pronouns or personal pronouns, but only refer to determiner phrases (DPs) (Abraham, 2002), and that d-pronouns prefer object antecedents (Bosch et al., 2003, 2007) and proto-patient antecedents (Schumacher et al., 2016). The current findings deviate dramatically from these characterizations.

### 3.3. Discussion–Corpus findings

The fact that the analysis of the *Tschick* corpus differs from previously assumed distributions of prominence-lending features with respect to the grammatical and thematic role of the antecedent might be primarily due to the dialogue structure of the *Tschick* corpus and its conversation-like narration style. Previous studies investigated prominence cues such as grammatical role and thematic role in referentially ambiguous contexts with two possible antecedents. In the *Tschick* corpus, however, not a single referentially ambiguous context is found for d-pronouns. The fact that nonetheless numerous d-pronouns occur in the corpus shows that the d-pronoun is not merely used to disambiguate referential conflicts and that its full function cannot be entirely attributed to prominence cues of the antecedent. Rather, the particular properties of the dialogue structure license the use of the d-pronoun: in direct speech, the respective passage is always communicated in the perspective of the person who utters the statement. As proposed by Hinterwimmer (2019, 2020), the choice of referential expression–especially the choice between a d-pronoun and a personal pronoun–is connected to the perspective holder. Specifically, the prominence scale of a d-pronoun is sensitive to perspective taking and hence the perspective holder represents the most prominent entity in a discourse. A d-pronoun can only be used to refer to an entity that is not the perspectival center of the passage. Hence a d-pronoun can be used to refer to a subject and agent antecedent if a perspective holder is available and if the referent that the d-pronoun is referring to is not the perspectival center. Thus, the surprising characteristics of the antecedents of the d-pronoun (being subjects/agents) reflects the prominent role of the perspectival center in the corpus.

## 4. The EEG experiment

### 4.1. Hypotheses

The aim of this study is to investigate how d-pronouns and personal pronouns are processed in larger naturalistic discourse contexts. To answer our research question, ERPs were recorded while participants listened to an audio book version of the previously annotated excerpt from the German novel *Tschick* (Herrndorf, 2010). Following Schumacher et al. (2015), we predict for the EEG experiment that processing the personal pronoun is rather effortless, while processing the d-pronoun is more costly. With regard to the electrophysiological components, (i) we hypothesize that referential expectations are derived from the prominence structure of the previous discourse, which has implication for the choice of referential expressions; deviations from these expectations are reflected in a more pronounced N400. In particular, the personal pronoun is considered to be the most continuous referential form, and the d-pronoun is a less expected referential form with respect to the maintenance of the prominence structure. Thus, we expect that the d-pronoun will evoke a distinct negative deflection around 400 ms in comparison to personal pronouns, because the d-pronoun is the more marked pronoun type and its usage is more unexpected compared to the personal pronoun. Additionally, (ii) we hypothesize that the d-pronoun signals attentional reorientation and this forward-looking function is reflected in the Late Positivity. In particular, we predict that the d-pronoun will show an enhanced late positive deflection relative to the personal pronoun, as a reflection of updating of the mental discourse representation triggered by attentional reorienting. Perspective-taking may contribute to such attentional reorientation. Originally, the hypotheses (i) and (ii) were the only ones we formulated prior to data collection and we expected that perspective-based effects might be reflected in the discourse updating and attentional orienting stage. But at this point, the results of the ERP experiments must be foreshadowed, since we found an additional perspective-related effect: The experiment elicited a sustained frontal positivity for d-pronouns relative to personal pronouns mirroring findings form perspective-taking. This effect was further scrutinized *post-hoc* and is described and interpreted in section 4.5 (*post-hoc* analysis of perspectival centers).

### 4.2. Method

#### 4.2.1. Participants

Forty-one participants (30 female, 11 male, 0 diverse) participated in the experiment. One participant was excluded due to a technical error during recording. The remaining participants' age ranged from 20 to 33 years (mean age 25.12 years, SD = 3.18) and all were monolingual native speakers of German. Thirty-five participants reported to not have read the novel nor have seen the movie *Tschick*. All participants gave written informed contest. They either received monetary compensation or course credits. Ethics approval for the study protocol was obtained from the ethics committee of the German Linguistic Society (#2016-09E2_200213).

#### 4.2.2. Material

The stimulus material of the EEG study is composed of the previously analyzed corpus of Wolfgang Herrndorf's novel *Tschick*. We used the official unshortened German audio book version read by Marius Clarén and published by the argon publishing company (Clarén, 2016). The nine chapters combined add up to 57:58 min of auditory presentation. The recording was segmented using automatic speech segmentation provided by the Munich Automatic Segmentation (MAUS) Web interface (Schiel, 1999; Kisler et al., 2016), combined with manual corrections at the onsets of the critical pronouns. The previously identified critical d-pronouns and personal pronouns from the corpus entered the analysis. Note however, that the *Tschick* corpus contains 144 critical personal pronouns, but the audio book version only included 143 critical personal pronouns, since one pronoun was realized as an “es” (it) instead of a “sie” (she) (*Neben ihm saß eine Frau und surfte die ganze Zeit im Internet, jedenfalls sah sie/es so aus*. “Next to him sat a woman, surfing the Internet the whole time, at least that's how she/it looked like.”).

#### 4.2.3. Procedure

**Figure 3:**
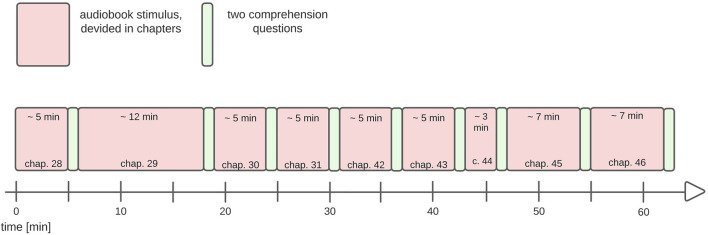
Presentation scheme. The total duration of the protocol was ~1 h. The audio book was presented in 9 consecutive parts with an average duration of 6:26 min per chapter. After each chapter, two comprehension questions were presented.

Participants sat in a soundproof booth in a comfortable chair. Before the experiment started, participants were instructed verbally, as well as in written instructions printed on the computer screen. The experiment was presented with the software Presentation. The auditory stimulus was presented *via* BOSE speakers (model: Companion 2). The volume was set equally for each participant at approximately 75 db. On a 24-inch computer screen, a fixation circle was visible during the presentation of the auditory stimulus. The light gray (Hex-code: #FAFAFA) fixation circle was depicted on a black (Hex-code: #000000) background. Participants held a controller in their hands that was used to answer comprehension questions after each chapter. Using the forefinger, a left and right button on the controller was pressed to indicate the intended answer corresponding to the answer options that were displayed on the left and right side of the screen. The comprehension questions merely served to keep the participants occupied during the experiment so that they would not lose their focus on the auditory input. Comprehension questions were not used to exclude trials or participants. Each experimental run consisted of nine chapters which correspond to nine different blocks. After each block, two comprehension questions were presented. Participants were instructed that they were allowed to move or stretch during the comprehension question phase. The mean block length was 6:26 min but the individual block lengths were as follows: the audio of chapter 28 took 5:41 min, chapter 29 took 12:09 min, the audios of chapter 30, 31, 42 and 43 were 5–6 min long (chap. 30: 5:51 min., chap. 31: 5:30 min., chapter 42: 5:06 min., chapter 43: 5:07 min.), chapter 44 took 3:13 min and the presentation of chapter 45 and 56 were approximately 8 min long (chap. 45: 7:36 min., chap. 46: 7:45 min.). In total 57:58 min of auditory input were presented. An experimental run took about an hour, but the duration could differ between participants, because there wasn't a set time frame for answering the comprehension question. Participants were able to take as much time as they wanted to answer the two comprehension questions. Figure 3 depicts a schematic description of the audio presentation.

#### 4.2.4. EEG recording and analysis

The electroencephalogram (EEG) was recorded from 64 Ag/AgCl scalp electrodes that were positioned according to the international 10–20 system on a flexible EEG cap (EasyCap, EasyCap GmbH, Herrsching, Germany). The ground electrode was placed at electrode position AFz. Further electrodes behind the left and right ear on the mastoid served for online referencing (left) and subsequent re-referencing of the EEG channels (right). Re-referencing was carried out offline after the completion of the experiment. To control for eye-movement artifacts, the electrooculogram (EOG) was recorded with additional eye electrodes that were placed to the left and right at the external canthus of each eye, as well as to the supra- and infra-orbital foramens of the left and right eye. The impendences of the electrodes were kept below 5 kΩ. All EEG and EOG channels were amplified with a Brain Products amplifier and recorded with a digitalization rate of 500 Hz. The data were analyzed using the MATLAB toolboxes EEGLAB (Delorme and Makeig, 2004), version 2021.0 and ERPLAB (Lopez-Calderon and Luck, 2014), version 8.10. First, independent component analysis (ICA) for artifact correction was calculated. For a better ICA decomposition, the EEG data were filtered with a 1 Hz high-pass filter and a 100 Hz low-pass filter to remove line noise. Then the data were re-referenced to linked mastoids and the EEG was filtered with a 0.6 Hz high-pass filter and a 30 Hz low-pass filter (Friederici et al., 2000; Wolff et al., 2008; Widmann et al., 2015; Maess et al., 2016a,b). Subsequently, artifact components computed by ICA were selected and removed from the filtered EEG; muscle and eye components above 80%, and heart components above 90% were removed. The ERPs were computed for a time window of 1,600 ms, starting 200 ms before the onset of the critical pronoun and lasting until 1,400 ms after stimulus onset.

#### 4.2.5. Data analysis

We calculated linear mixed-effect models using the lmerTest package (Kuznetsova et al., 2017) in RStudio (version 1.4.110; RStudio Team, 2021) with amplitude as dependent variable. The models included the fixed effect PRONOUN (personal pronoun/d-pronoun) as well as the two continuous topographic fixed effects SAGGITALITY and LATERALITY, which are based on the coordinates of the standard BESA coordinate system, and all interactions between them. We calculated the models for a time window from 0 to 1,400 ms after pronoun onset in steps of 100 ms. The model was fitted using a backward approach, starting with maximally specified random effects and subsequently minimizing the model until it would converge (e.g., Barr et al., 2013). We then used the maximal model that converged in every time window. For each time window the model included a by-participant intercept and by-participant random slopes for each fixed factor without interactions as well as by-item varying intercepts. A more complex model adding random slopes for item would not converge in every time window. The model we calculated for each time window is shown in (3):

(3) lmer (uV ~ saggitality ^*^ laterality ^*^ pronoun + (1 + saggitality + laterality + pronoun | subject) + (1 | item))

### 4.3. Results

**Figure 4:**
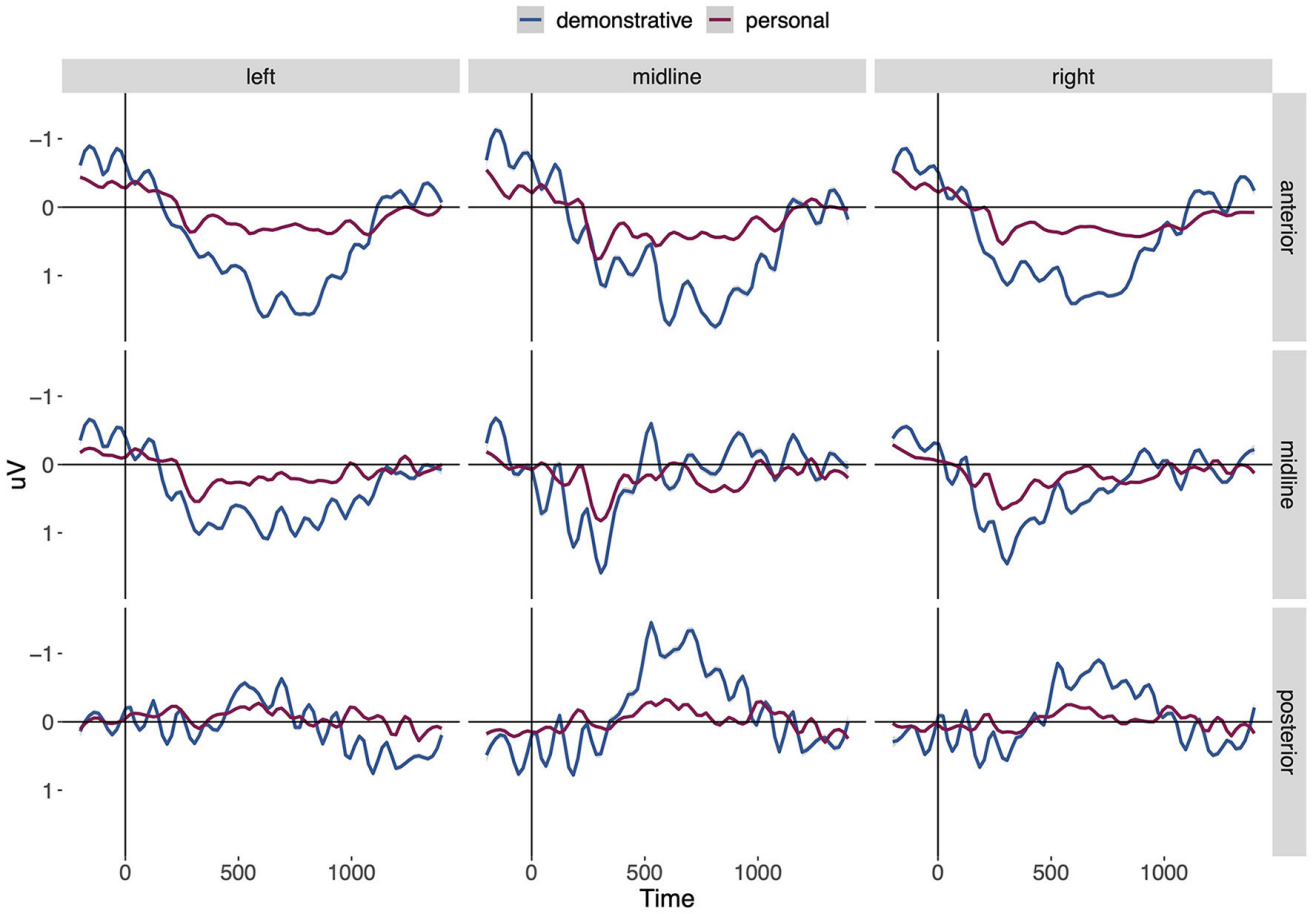
Grand-average ERPs for d-pronouns (blue) and personal pronouns (red) averaged for nine regions of interest. The vertical line indicates the onset of the pronouns. Negativity is plotted up.

Figure 4 shows the mean amplitudes of the ERPs over time by condition and ROI. As becomes clear from Figure 4, our results reveal a biphasic processing pattern at posterior electrodes for the d-pronoun in comparison to the personal pronoun. Looking at the time course, the data show an enhanced negative deflection starting around 300 ms, followed by a more pronounced positivity from 1,000 to 1,400 ms. In addition to the biphasic processing pattern with its posterior distribution, the figure reveals a pronounced positivity at anterior (and left lateral) electrodes for the d-pronoun relative to the personal pronoun between 500 and 1,200 ms.

Based on an initial 100 ms time window analysis, we determined larger time windows involving effects of PRONOUN that span across at least 2 successive windows and grouped them by their interaction with the topographical factors SAGGITALITY and LATERALITY. In this way, three larger effect windows were determined, spanning from 300 to 1,000 ms (building on a SAGGITALITY^*^PRONOUN interaction), from 1,000 to 1,400 ms (where the estimate of the SAGGITALITY^*^PRONOUN interaction shows another sign), and from 500 to 1,200 ms (with a LATERALITY^*^PRONOUN interaction).

For the 300–1,000 ms effect window, the statistical analysis revealed a significant two-way interaction SAGGITALITY^*^PRONOUN, which indicates that the negative deflection for d-pronouns relative to personal pronouns has a posterior distribution. The subsequent interaction of SAGGITALITY^*^PRONOUN between 1,000 and 1,400 ms strengthens the posterior maximum of the later positivity. For the 500–1,200 ms effect window, the significant two-way interaction of LATERALITY^*^PRONOUN (together with the SAGGITALITY^*^PRONOUN interaction spanning across this time window) points to the anterior and left lateral distribution of an additional positive deflection for the d-pronoun. Estimates, *t-*values and *p-*values of the 100 ms time window analyses are given in Table 1 grouped by the three effect windows. To consider the problem of multiple comparisons, we also report a Bonferroni-corrected *p-*value, based on the 14 time windows considered in the analysis, yielding *p* < 0.0036.

**Table 1:** Summary of significant interactions in the main time windows.

**Effect**	**100 ms time windows**	**Estimate**	**t**	***p***
**Saggitality** ^*^pronoun (300–1,000 m)	300–400 ms	−0.161	−5.831	5.51 × 10^−9^^***^
	400–500 ms	−0.302	−10.725	<2 × 10^−16^^***^
	500–600 ms	−0.484	−17.363	<2 × 10^−16^^***^
	600–700 ms	−0.588	−21.867	<2 × 10^−16^^***^
	700–800 ms	−0.594	−20.391	<2 × 10^−16^^***^
	800–900 ms	−0.452	−13.452	<2 × 10^−16^^***^
	900–1,000 ms	−0.231	−7.361	1.82 × 10^−13^^***^
**Laterality** ^*^pronoun (500–1,200 ms)	500–600 ms	0.053	2.019	4.34 × 10^−2^^*^
	600–700 ms	0.073	2.884	3.93 × 10^−3^^*^
	700–800 ms	0.179	6.526	6.75 × 10^−11^^***^
	800–900 ms	0.222	7.014	2.32 × 10^−12^^***^
	900–1,000 ms	0.209	7.042	1.90 × 10^−12^^***^
	1,000–1,100 ms	0.085	3.179	1.479 × 10^−3^^**^
	1,100–1,200 ms	0.064	2.342	1.92 × 10^−2^^*^
**Saggitality** ^*^pronoun (1,000–1,400 ms)	1,000–1,100 ms	0.0999	3.542	3.97 × 10^−4^^***^
	1,100–1,200 ms	0.224	7.710	1.27 × 10^−14^^***^
	1,200–1,300 ms	0.218	7.320	2.48 × 10^−13^^***^
	1,300–1,400 ms	0.262	8.913	<2 × 10^−16^^***^

Significance coding: “^***^” < 0.001, “^**^” < 0.0036, “^*^” < 0.05.

### 4.4. Discussion–ERP findings

Studies on pronoun resolution have mostly utilized short texts consisting of a context and a target sentence. In the current study we presented participants with nine chapters of an audio book while recording their EEG to investigate the real-time resolution of personal and demonstrative pronouns in a more ecologically valid setting. We expected to see more demanding processing costs for the d-pronoun in comparison to the personal pronoun. And indeed, the current ERP study replicated previous findings from the processing of d-pronouns that showed a biphasic N400-Late Positivity pattern. Crucially, it revealed an additional sustained anterior positivity for d-pronouns.

Looking at posterior electrodes, in the 300–1,000 ms time window, the d-pronoun shows an enhanced negativity compared to the personal pronoun. This negativity can be described as an N400 effect, which varies depending on the degree of probability and expectation (Kutas and Hillyard, 1983; Schumacher et al., 2015). In the 1,000–1,400 ms time window, the results reveal a positive deflection for d-pronouns over personal pronouns. This positivity is here taken to be a Late Positivity, which is indicative of increased processing costs caused by attentional orienting and the updating of the mental discourse model (Burkhardt, 2005; Schumacher et al., 2015). Its time course is somewhat delayed compared to previously reported updating effects, which we attribute to the particular nature of the audio book stimuli. During the processing of an entire audio book plot, a complex mental model (involving numerous individuals, events, time points and relations between these entities) has to be constructed and maintained continuously for the unfolding story, i.e., the cognitive load associated with discourse management and updating is higher than in two-sentence scenarios that have typically been used in previous lab experiments.

Overall, the results reveal a biphasic N400–Late Positivity pattern at posterior electrodes for the d-pronoun relative to the personal pronoun, thereby confirming previous findings with highly controlled stimuli (Schumacher et al., 2015). We take the observed N400 for the d-pronoun as an indication for more demanding processing costs that occur due to the relative unexpectedness of this referential expression and its markedness in comparison to the personal pronoun. The Late Positivity is taken to reflect the consequences of attentional reorientation: since the d-pronoun indicates a possible shift in the discourse structure with respect to the ranking of the referential candidates, it induces updating of the discourse structure.

In addition to the biphasic pattern, the data showed an enhanced positivity at anterior electrode sites for the d-pronoun relative to the personal pronoun. We suggest that this frontal positivity reflects another attention-based signal triggered by the d-pronoun and is specifically linked to perspective taking since the use of the d-pronoun in this particular novel is often tied to the evaluation by the perspective holder. To further understand the nature of the frontal positivity, we ran a *post-hoc* analysis with the additional factor perspectival center, which is reported immediately below.

### 4.5. *Post-hoc* analysis of perspectival centers

#### 4.5.1. Data analysis

To determine whether the observed frontal positivity for d-pronouns is related to changes of perspective between the narrator (Maik) and other prominent perspective holders (Tschick or Maik's father), a *post-hoc* analysis involving perspective takers was performed for the d-pronouns. First, we divided the instances of the critical d-pronouns into three categories of perspective holders: Maik/Narrator (*n* = 11), Tschick (*n* = 12), and Father (*n* = 10). When a d-pronoun occurred in direct speech, it was assigned to the perspective holder or speaker of this speech act. D-pronouns that occurred in parts that are narrated by the narrator were assigned to the perspective of the narrator (i.e., Maik). We also found two instances where a d-pronoun is uttered in the protagonist Maik's direct speech. Since the protagonist Maik is the narrator of the novel, we decided to collapse all d-pronouns that occur in neutral narrated parts and those that occur in direct speech parts uttered by Maik/the narrator. Note that the great majority (90.61%, *n* = 164) of all personal pronouns are rendered by the narrator of the novel, hence a perspective shift only occurs in <10% of cases.

Subsequently, we calculated a similar mixed-effect model as described in section 4.2.5, but instead of the fixed effect PRONOUN we used the fixed effect PERSPECTIVE (Maik, Tschick, Father) as well as the two continuous topographic fixed effects SAGGITALITY and LATERALITY. The analysis included a by-participant intercept and by-participant random slopes for each fixed factor without interactions, as well as by-item varying intercepts. The data reported are based on the model shown in (4).

(4) lmer (uV ~ saggitality ^*^ laterality ^*^ perspective + (1 + saggitality ^*^ laterality ^*^ perspective | subject) + (1 | item))

We calculated the model for the time window of the frontal positivity (500–1,200 ms) observed in the overall analysis reported above.

#### 4.5.2. Results

**Figure 5:**
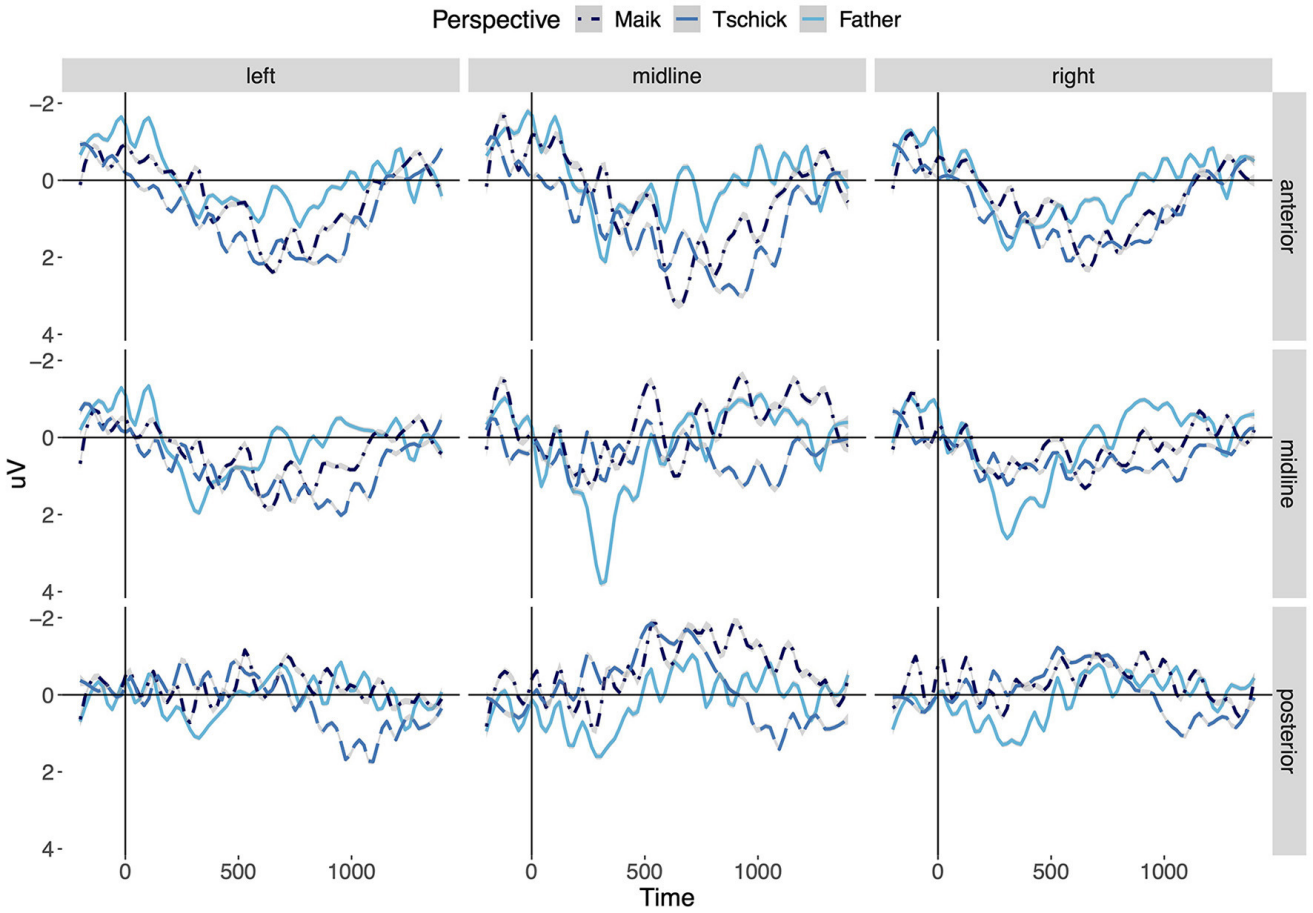
Grand-average ERPs of d-pronouns comparing three perspective takers: Maik/narrator (dark blue, dotdashed line), Tschick (blue, dashed line), and father (light blue, solid line). The vertical line indicates stimulus onset.

The *post-hoc* analysis of perspective holders revealed distinct processing differences between the three perspectival centers Tschick, Maik and the Father. The ERPs at anterior electrodes show a clear gradation between the categories. D-pronouns that expressed the stance of protagonist Tschick elicit the most pronounced positivity, followed by d-pronouns that express the perspective of the narrator Maik. The most reduced positivity is observed for d-pronouns that express the stance of the narrator's father. These findings are visualized in Figure 5, which depicts the mean amplitudes of the ERPs of d-pronouns over time by perspective holders and regions of interest.

Table 2 shows the significant interactions of perspective and region of interest, indicative of the anterior effect. The time window shows a significant interaction of SAGGITALITY^*^PERSPECTIVE and LATERALITY^*^PERSPECTIVE. A gradation between all three perspective holders can be observed in the time range between 500 and 1,200 ms. D-pronouns uttered in the stance of the Father differ significantly from d-pronouns uttered in the stance of Tschick as well as from d-pronouns uttered in the stance of Maik.

**Table 2:** Overview of significant interactions with the factor perspective.

**Time window**	**Effect**	**Estimate**	**t**	***p***
**500–1,200 ms**	saggitality*perspective [Maik]	0.242	5.042	4.63 × 10^−7***^
	saggitality*perspective [Tschick]	0.187	3.993	6.53 × 10^−5***^
	laterality*perspective [Tschick]	−0.159	−3.593	3.27 x 10^−4***^

Significance coding: “^***^” <0.001, “^**^” <0.01, “^*^” <0.05.

#### 4.5.3. Discussion–*Post-hoc* ERP analysis

The *post-hoc* analysis of perspective holders of the d-pronouns revealed a graded frontal positive effect between the three perspectival centers emerging in the narrative. D-pronouns expressing the perspective of Tschick show the most pronounced positivity followed by d-pronouns in the stance of Maik followed by d-pronouns anchored by the father. Interestingly, the ERPs of d-pronouns uttered by the two main protagonists show distinctly stronger positivities than d-pronouns uttered by the father. The two main protagonists of the youth novel under investigation can be described as brave, cool, rebellious, free, and spontaneous and thus represent very likable and identifiable protagonists. The father, on the other hand, represents a mean, unfair, not caring and lying character that is set to be the antagonist of the main characters. Therefore, we suggest that listeners of the audio book engage less with the perspective of the father than with the perspective of the two main protagonists. The pronounced frontal positivity for d-pronouns uttered in the stance of Tschick and Maik is thus taken to reflect enhanced identification with these two characters. This can be related to results of Brilmayer et al. (2019), underlining the suggestion that main protagonists cause stronger effects due to a stronger level of engagement with these protagonists. However, the study by Brilmayer et al. (2019) compared to which referent the pronoun under investigation was referring to (main protagonist vs. other protagonist), while in our analysis, we looked at which protagonist's (main protagonist vs. other protagonist) perspective is rendered by the d-pronoun. From research on perspective taking it seems, though, that the shift in perspective is not the only factor causing the observed pattern. According to the approach by Harris (2021) the default perspective holder should be the narrator and a shift from this perspective should elicit enhanced processing costs (for costs of shifting see Sabbagh and Taylor, 2000; Zhou et al., 2010; Meinhardt et al., 2011; Shi et al., 2011; Richter et al., 2020). In Figure 5, however, we see the tendency that d-pronouns that render the narrator's perspective cause comparatively high processing costs in form of the frontal positivity. In contrast, d-pronouns that render the perspective of the father show a relatively reduced frontal positivity. Hence, we assume that the factor of main protagonists vs. other protagonists rather has an influence on the processing costs of d-pronouns that is due to the willingness of the reader to engage with the perspective of the protagonist.

## 5. General discussion

The present study used naturalistic stimuli from an audio book to examine the processing of d-pronouns compared to personal pronouns. The prevalent dialogue structure in the novel *Tschick* allowed us to investigate referential resolution in a dynamic communication situation that licensed the use of the demonstrative pronoun and also made available the perspectival center as a prominence-lending cue. The notion of perspective as a particular feature of the stimuli yielded two critical findings, which have not been reported for classical lab experiments: first, the corpus analysis revealed an overwhelming subject/agent antecedent choice of the d-pronoun (resembling the interpretive pattern observed for the personal pronoun). Second, the ERP data generated an additional frontal positivity effect, indicative of perspective-based attention orienting triggered by the d-pronoun.

### 5.1. Neural correlates of d-pronouns

Our first aim was to investigate pronoun resolution in a more ecologically valid scenario by recording ERPs while listening to an audio book. This was motivated by a general desire to overcome potential communicative limitations of highly controlled lab experiments, but also by caveats concerning the likelihood of encountering a d-pronoun in certain contexts, since d-pronouns have been associated with informal and oral communication in traditional grammars (see Patil et al., 2020). Turning to a coming-of-age novel with colloquial dialogues between two teenagers dismissed this concern.

Previous research reported effects for two core functions of d-pronouns–expectation-based processing and discourse updating (Schumacher et al., 2015)–which were also observed in the present study. D-pronouns showed a more pronounced N400, indicating that the d-pronoun in comparison to the personal pronoun is a less expected and more marked referential form, given expectations derived from the prominence ranking of referents in the current discourse and general constraints on coherence (i.e., maintain the currently most prominent referential entity; Grosz et al., 1995). Crucially, while previous research was undecided whether this effect was due to the encountering of a less expected form (personal pronouns are more predicted than d-pronouns) or prominence-based expectations (associated likelihood for less prominent antecedent), the fact that the current corpus data indicate very similar resolution preferences for the two types of pronouns suggests that the current N400 is mostly driven by form-specific expectations.

Moreover, d-pronouns evoked a Late Positivity, which has been associated with the updating of the mental representation based on the demonstrative's forward-looking function indicating that the respective referent may become more prominent in subsequent discourse (Abraham, 2002; Fuchs and Schumacher, 2020). In addition to confirming findings from traditional lab experiments, the current study registered an extra frontal positivity, which we attribute to perspective-based operations. This will be elaborated in the next section.

### 5.2. D-pronouns and perspectival centers

The most intriguing results of the current study are intimately intertwined with perspective taking. At first sight, the corpus analysis brought force a surprising interpretive behavior of the third person d-pronouns, that is that they overwhelmingly prefer a subject and agent antecedent (see Figure 2). In this respect, they do not differ from the personal pronouns in the corpus. This stands in stark contrast to the assumption that demonstrative pronouns follow an anti-subject, anti-agent, and/or anti-topic strategy in selecting their antecedent (e.g., Bosch et al., 2003, 2007; Bosch and Umbach, 2007; Kaiser and Trueswell, 2008; Kaiser, 2011b; Schumacher et al., 2016). However, it is compatible with Hinterwimmer's proposal that perspectival centers represent maximally prominent referents for d-pronouns yielding an avoidance of the perspectival center and selection of the next prominent entity, which is the subject/agent (Hinterwimmer and Bosch, 2016, 2017; Hinterwimmer, 2019). The audio book corpus thus corroborates Hinterwimmer's proposal and allowed us to take into consideration the notion of perspectival centers for the real-time investigation of d-pronouns.

While the availability of perspectival centers as prominence-lending features did not have a major impact on the biphasic N400-Late Positivity pattern (in as far as the patterns resembled earlier studies that did not manipulate perspective), we interpret the additional frontal positivity as a reflex of perspective-based processing. The positivity is most pronounced for utterances anchored to the two teenage protagonists (the narrator Maik and his friend Tschick vs. Maik's father), which suggests to us a certain degree of engagement and identification with these two protagonists. This may be another instance of processing self-relevant stimuli, which engenders a positivity (e.g., Gray et al., 2004; Knolle et al., 2013). The use of a d-pronoun may also coincide with an expressive function, carrying a derogatory connotation. Expressive content has been associated with a later positive-going wave (e.g., Donahoo and Lai, 2020 on swear words). However, a comparison of expressively used d-pronouns (*n* = 20) with non-expressive d-pronouns (*n* = 13) from our stimulus set registered no observable differences. We thus conclude that the frontal positivity is primarily triggered by identification with the perspective holder.

## 6. Conclusion

In sum, our study presents novel insights regarding pronoun resolution in naturalistic texts. We were able to replicate a biphasic processing pattern for d-pronouns, which was previously observed in highly controlled ERP experiments. Moreover, we found an additional effect in form of a large frontal positivity for d-pronouns, which we assume to be related to the perspectival centers with gradient differences between the main protagonists who listeners engage with and other protagonists. This indicates that the particular attention orientation of the d-pronoun in terms of perspective taking may unfold its function more naturally within the naturalistic context. Thus, by using naturalistic stimuli, we get one step closer to understanding the implementation of language processing in the brain during real-life language comprehension.

## Data availability statement

The raw data supporting the conclusions of this article will be made available by the authors, without undue reservation.

## Ethics statement

The studies involving human participants were reviewed and approved by Ethics Board of the German Linguistic Society. The patients/participants provided their written informed consent to participate in this study.

## Author contributions

MR: investigation, data curation, formal analysis, visualization, writing—original draft, writing—review, and editing. PS: conceptualization, methodology, investigation, formal analysis, writing—original draft, writing—review and editing, supervision, project administration, and funding acquisition. Both authors contributed to the article and approved the submitted version.

## Footnotes

^1^Although we only annotated animate REs, we find 3 pronouns with neuter gender. The neuter personal pronoun goes back to a passage in the novel where the main protagonists discover a suspicious shadow. However, the reader can already assume at this point that the shadow refer to the acquaintance Isa. Thus, the neutral pronoun in fact refers to an animate entity. The two neuter d-pronouns are used in pejorative constructions of situational deixis (e.g., *Was ist das (DEM) denn für ein Idiot*?, “What kind of idiot is that?”).
